# Subject-specific one-dimensional fluid dynamics model of chronic thromboembolic pulmonary hypertension

**DOI:** 10.1007/s10237-023-01786-3

**Published:** 2023-11-29

**Authors:** Amirreza Kachabi, Mitchel J. Colebank, Naomi C. Chesler

**Affiliations:** Edwards Lifesciences Foundation Cardiovascular Innovation and Research Center, Department of Biomedical Engineering, University of California, Irvine, Irvine, CA USA

**Keywords:** CTEPH, 1D CFD, Hemodynamics modeling, Wall shear stress

## Abstract

Chronic thromboembolic pulmonary hypertension (CTEPH) develops due to the accumulation of blood clots in the lung vasculature that obstructs flow and increases pressure. The mechanobiological factors that drive progression of CTEPH are not understood, in part because mechanical and hemodynamic changes in the small pulmonary arteries due to CTEPH are not easily measurable. Using previously published hemodynamic measurements and imaging from a large animal model of CTEPH, we applied a subject-specific one-dimensional (1D) computational fluid dynamic (CFD) approach to investigate the impact of CTEPH on pulmonary artery stiffening, time-averaged wall shear stress (TAWSS), and oscillatory shear index (OSI) in extralobar (main, right, and left) pulmonary arteries and intralobar (distal to the extralobar) arteries. Our results demonstrate that CTEPH increases pulmonary artery wall stiffness and decreases TAWSS in extralobar and intralobar arteries. Moreover, CTEPH increases the percentage of the intralobar arterial network with both low TAWSS and high OSI, quantified by the novel parameter $\varphi$, which is related to thrombogenicity. Our analysis reveals a strong positive correlation between increases in mean pulmonary artery pressure (mPAP) and $\varphi$ from baseline to CTEPH in individual subjects, which supports the suggestion that increased $\varphi$ drives disease severity. This subject-specific experimental–computational framework shows potential as a predictor of the impact of CTEPH on pulmonary arterial hemodynamics and pulmonary vascular mechanics. By leveraging advanced modeling techniques and calibrated model parameters, we predict spatial distributions of flow and pressure, from which we can compute potential physiomarkers of disease progression. Ultimately, this approach can lead to more spatially targeted interventions that address the needs of individual CTEPH patients.

## Introduction

Chronic thromboembolic pulmonary hypertension (CTEPH) occurs when blood clots lodge in the small blood vessels of the lung and fail to fully dissolve or resolve over time. The resulting blood flow obstruction leads to an elevation in blood pressure within the pulmonary circulation. If left untreated, CTEPH causes right ventricular failure and death. With early diagnosis, however, CTEPH can be successfully treated with surgery (Feinstein et al. 2001). Pulmonary endarterectomy is the primary treatment choice for individuals with proximal clots and frequently results in a successful recovery. A treatment option for patients with clots located further downstream in smaller (intralobar) pulmonary arteries is balloon pulmonary angioplasty. CTEPH is diagnosed by invasive right heart catheterization (RHC) to measure mean pulmonary artery pressure (mPAP) ≥ 20 mmHg and pulmonary capillary wedge pressure (PCWP) ≤ 15 mmHg, with subsequent imaging to confirm clot burden (Lang and Madani 2014).

A key knowledge gap in CTEPH is the drivers of clot growth and dissolution over time (Tsubata et al. 2023). Vascular mechanobiological factors are thought to play an important role. Abnormalities in the magnitude and directions of wall shear stress (WSS) contribute to pathological processes such as endothelial dysfunction, inflammation, and impaired vasodilation (Allen et al. 2023). WSS in intralobar pulmonary arteries likely impacts clot organization and growth as well as dissolution (Tsubata et al. 2023). Given that pulmonary artery stiffness increases with pulmonary hypertension development (Wang and Chesler 2011) and alters flow and thus shear stress dynamics (Su et al. 2013), pulmonary artery stiffness may also contribute to CTEPH progression. Therefore, computing WSS and vascular mechanics throughout the pulmonary arterial network can provide useful information regarding likely drivers of disease progression.

Large animal models of CTEPH have advanced our understanding of the pathophysiology of CTEPH (Mulchrone et al. 2019; Stam et al. 2021), but typically do not report local mechanical stimuli for disease progression such as WSS. Image-based computational fluid dynamics (CFD) modeling can compute pulmonary artery blood flow with high spatial resolution (Burrowes et al. 2017; Bordones et al. 2018; Wang et al. 2020), from which WSS can be calculated. The combination of experiments in large animal models of CTEPH with CFD analysis enables estimation of mechanobiological stimuli in sections of the pulmonary arterial network inaccessible to measurement. Moreover, this combination of techniques can be used to compute stimuli that cannot be measured from imaging alone, such as pressure distribution.

Here, we use an image-based nonlinear one-dimensional (1D) CFD model to predict hemodynamics throughout the pulmonary arterial network at baseline and after experimentally-induced CTEPH. The large animal data were previously published by Mulchrone et al. (2019). We calibrate our CFD model to the hemodynamic and imaging data available and then use the results to quantify the impact of CTEPH on metrics of WSS including time-averaged WSS (TAWSS) and oscillatory shear index (OSI). We also quantify the impact of CTEPH on wall stiffness.

Our objective was to leverage clinically measurable hemodynamic data and imaging to forecast non-clinically measurable hemodynamic and mechanical parameters relevant to pulmonary vascular disease progression. We used imaging data to build subject-specific geometries of the pulmonary arterial network for fluid dynamics simulation of healthy and diseased states. After calibrating the model to measured hemodynamics, we performed 1D model simulations to investigate the impact of CTEPH on spatial and temporal distributions of wall shear stress and vascular mechanics. This synergistic experimental–computational framework gives insight into the extralobar and intralobar mechanobiological effects of CTEPH with subject specificity and provides a future tool for understanding the local determinants of disease progression seen clinically.

## Methods

### Animal study

All experimental data were obtained retrospectively; a detailed protocol and complete reporting of measurements can be found in Mulchrone et al. (2019). Briefly, CTEPH was induced by injecting multiple microspheres into the pulmonary arteries (PAs) using an indwelling catheter in five adult male canines (12 ± 1 kg body weight) following methods established by Hori et al. (2012). Magnetic resonance imaging (MRI) and RHC were performed under anesthesia sustained by administering 1–3% isoflurane in 100% oxygen, and ventilation regulated to maintain end-tidal CO_2_ levels within the range of 30–50 mmHg. Instrumentation details are available in Bellofiore et al. (2013). In our analysis of the images from the original study by Mulchrone et al. (2019), there was no evidence of proximal (MPA, LPA, or RPA) occlusion after CTEPH induction.

### Hemodynamic and imaging data

All measured hemodynamic data were collected at baseline and after animals reached an mPAP ($\overline{P}_{{{\text{PA}}}}$) ≥ 25 mmHg (based on clinical guidelines when these experiments were conducted (Lang and Madani 2014)) and mean PCWP ($\overline{P}_{{{\text{PCW}}}}$) ≤ 15 mmHg as detailed in Bellofiore et al. (2013). Pressure data included systolic and diastolic main pulmonary artery (MPA) pressures and mean PCWP. Imaging data, including time-series velocity and area in the MPA, left (LPA), and right (RPA) pulmonary arteries (i.e., the extralobar PAs), were obtained from phase-contrast MRI. Time-series flow, $Q\left( t \right)$ (mL/s), was calculated as the product of velocity and cross-sectional area for each extralobar PA. MR angiography was also performed with contrast to visualize the pulmonary arterial network geometry.

### CFD model geometry

**Fig. 1:**
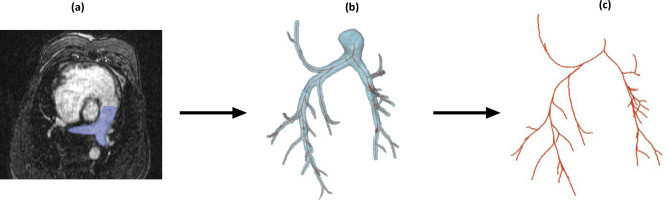
Process for extracting 1D arterial network for one subject at baseline, including **a** arterial identification from MRI images, **b** 3D segmentations of the arterial network, and **c** network extraction obtained by conversion from the 3D segmentations

We created a 1D arterial network model as the computational domain for each subject at baseline and with CTEPH, shown for a representative subject at baseline in Fig. 1. First, from time-averaged MR angiography images (Fig. 1a), 3D segmentations of extralobar and intralobar PA geometries were created using 3D slicer (Fedorov et al. 2012) (Fig. 1b). We then used the vascular Modeling Toolkit to convert the 3D segmentations to 1D centerline networks (Fig. 1c) (Antiga et al. 2003). Finally, we post-processed the specific networks using custom MATLAB (Natick, MA) software to create the 1D CFD mathematical domain for simulations (Colebank et al. 2021). Note, MR angiography and phase-contrast images at the MPA, RPA, and LPA were not recorded simultaneously. Therefore, we scaled the extralobar PA area in the network geometry to match the phase-contrast data at diastole since the reference area for the pressure–area relationship is chosen to be the diastolic area.

### Governing equations

We use time-dependent, 1D fluid dynamics equations to simulate hemodynamics in the pulmonary arterial networks. We assume that all blood vessels are straight, cylindrical, and impermeable. Blood flow through the arterial network is assumed to be axisymmetric, incompressible, Newtonian, and laminar such that the governing equations are as follows:

1 $$ \frac{\partial Q}{{\partial x}} + \frac{\partial A}{{\partial t}} = 0 $$

2 $$ \frac{\partial Q}{{\partial t}} + \frac{{\left( {\gamma + 2} \right)}}{{\left( {\gamma + 1} \right)}}\frac{\partial }{\partial x}\left( {\frac{{Q^{2} }}{A}} \right) + \frac{A}{\rho }\frac{\partial P}{{\partial x}} = - \frac{{2\pi \mu \left( {\gamma + 2} \right)}}{\rho }\frac{Q}{A} $$

where $x$ (cm) and $t$ (s) represent the axial and temporal coordinates, and $P\left( {x,t} \right)$ is the transmural blood pressure (mmHg) (Olufsen et al. 2000). $Q\left( {x,t} \right)$ is the volumetric flow (mL/s), and $A\left( {x,t} \right) = \pi \left( {r\left( {x,t} \right)} \right)^{2}$ is cross-sectional area (cm^2^) where $r\left( {x,t} \right)$ is the spatially- and temporally-dependent radius of the artery (cm). The density ($\rho$) and dynamic viscosity ($\mu$) of blood are assumed to be constant and equal to 1.03 (g/mL) and 0.03 (Poise), respectively (Kamiya and Togawa 1980). Further, we assume that the fluid velocity at the wall is equal to the velocity of the wall (no-slip condition).

**Fig. 2:**
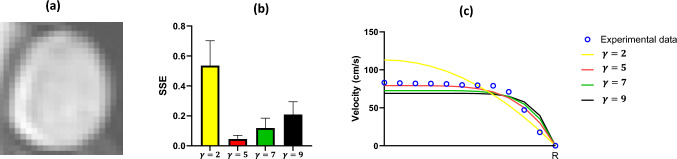
**a** A snapshot of temporal 2D phase image of a subject in baseline at systole. **b** A bar graph of mean and standard deviation of sum of square error (SSE) for all subjects in baseline and CTEPH with $\gamma = 2, 5, 7,$ and $9$. **c** Matching experimental data with power-law velocity profiles with different powers for this subject in baseline

The 1D theory requires an explicit expression for the fluid velocity profile. Here, we use the power-law profile:

3 $$ u\left( {x,t} \right) = \overline{U}\left( t \right)\frac{\gamma + 2}{\gamma }\left( {1 - \frac{{r\left( {x,t} \right)^{\gamma } }}{{R^{\gamma } }}} \right) $$

where $u\left( {x,t} \right)$ is the blood velocity (cm/s), $\overline{U}\left( t \right)$ is the mean velocity (cm/s), $R$ is radius of the artery (cm), and $\gamma$ is the power-law constant. To determine the most suitable $\gamma$ value for the inlet velocity profile, we compared the measured velocity profile to the assumed power-law velocity profiles. Figure 2a shows a representative 2D phase-contrast image of the MPA of a subject at baseline for peak systole. To obtain the measured velocity profile, we averaged the image intensity values across eight sections that spanned the radius from the wall to the lumen center (every 45°). We then computed the flow rate for this profile over the cross-sectional area, and for the same flow rate and zero velocity at the wall (peak velocity at the center), we generated power-law profiles for $\gamma = 2, 5, 7,$and $9$. With the caveat that image resolution near the wall is limited, $\gamma = 5$ provided the best fit (lowest residual sum of squares) for all subjects at both baseline and CTEPH (Fig. 2b). Thus, $\gamma = 5$ was used for in the power-law velocity profile for all subjects. Figure 2c shows the experimental data and flow rate-matched power-law velocity profiles with $\gamma = 2, 5, 7,$ and $9$ for a subject in baseline.

### Wall mechanics

Within the given 1D equations, there are three dependent variables, $\left\{ {P\left( {x,t} \right),A\left( {x,t} \right), Q\left( {x,t} \right)} \right\}$. To close the system of equations, a constitutive model for the artery wall is required. We used a linear elastic model (Safaei et al. 2016), which assumes a linearly elastic, thin-walled, isotropic, and incompressible material in the form of a cylinder. Under these circumstances, the constitutive law can be written as follows:

4 $$ P\left( {x,t} \right) = \frac{4}{3}\frac{Eh}{{r_{0} }}\left( {\sqrt {\frac{{A\left( {x,t} \right)}}{{A_{{{\text{dia}}}} }}} - 1} \right) + P_{{{\text{dia}}}} $$

where $E$ is the circumferential Young’s modulus (mmHg), $h$ (cm) is the wall thickness, and $A_{{{\text{dia}}}} = \pi r_{{{\text{dia}}}}^{2}$ (cm^2^) is the lumen area at the diastolic pressure $P_{{{\text{dia}}}}$ (mmHg). The term $\frac{Eh}{{r_{0} }}$ accounts for both structural and load-dependent stiffening and is a potential physiomarker of disease severity. We calculated this term analytically for individual subjects at baseline and CTEPH using systolic pressure and area in the MPA in Eq. (4). Finally, model equations were solved using two-step Lax–Wendroff method, detailed in prior work (Olufsen et al. 2000).

### Boundary conditions

From the set of measurements mentioned earlier, the following were used as input: $\left\{ {\overline{P}_{{{\text{PA}}}} , \overline{P}_{{{\text{PCW}}}} , Q_{{{\text{MPA}}}} \left( t \right)} \right\}$, and the following were used for calibration: $\left\{ {P_{{{\text{sys}}}} , P_{{{\text{dia}}}} , A_{{{\text{sys}}}} , A_{{{\text{dia}}}} , Q_{{{\text{LPA}}}} \left( t \right), Q_{{{\text{RPA}}}} \left( t \right)} \right\}$, where $P_{{{\text{sys}}}} , A_{{{\text{sys}}}} , P_{{{\text{dia}}}} ,$ and $A_{{{\text{dia}}}}$ are the pressures and areas in the MPA at systole and diastole, respectively, $\overline{P}_{{{\text{PA}}}} = 1/3P_{{{\text{sys}}}} + 2/3P_{{{\text{dias}}}}$ is the mean MPA pressure, and $Q_{{{\text{MPA}}}} \left( t \right),Q_{{{\text{LPA}}}} \left( t \right)$, and $Q_{{{\text{RPA}}}} \left( t \right)$ are the MPA, LPA, and RPA flows, which are functions of time. The time-averaged MPA flow, $\overline{Q}_{{{\text{MPA}}}}$, is also used with $\overline{P}_{PA}$ and $\overline{P}_{{{\text{PCW}}}}$ to compute overall pulmonary vascular resistance as ${\text{PVR}} = \left( {\overline{P}_{{{\text{PA}}}} - \overline{P}_{{{\text{PCW}}}} } \right)/\overline{Q}_{{{\text{MPA}}}}$. Boundary conditions are specified at each vessel inlet and outlet. We used the $Q_{{{\text{MPA}}}} \left( t \right)$ as the inlet flow boundary condition in the MPA. At extralobar and intralobar vessel junctions, flow conservation $Q_{{\text{p}}} = Q_{{{\text{d}}1}} + Q_{{{\text{d}}2}}$ and pressure continuity $P_{{\text{p}}} = P_{{{\text{d}}1}} = P_{{{\text{d}}2}}$ were enforced, where the subscript ‘*p*’ denotes the parent vessel that bifurcates into two daughter vessels ‘*d*_1_’ and ‘*d*_2_.’

At each terminal intralobar vessel, we used three element Windkessel models to represent all vessels outside those obtained by segmentation. Each Windkessel model includes a proximal resistance $$\left( {R_{{\text{P}}} } \right)$$, distal resistance $$\left( {R_{{\text{D}}} } \right)$$, and compliance $$\left( C \right)$$. Total nominal Windkessel resistance of each terminal vessel is calculated based on the pressure difference and mean flow in that vessel, $$R_{{{\text{T}}\left( i \right)}} = \left( {P_{{{\text{mean}}}} - P_{{{\text{PCW}}}} } \right)/\overline{Q}_{i} = R_{{{\text{P}}\left( i \right)}} + R_{{{\text{D}}\left( i \right)}}$$, where $$\overline{Q}_{i}$$ is the mean flow to vessel $$i $$ determined by applying Poiseuille argument at each junction that gives

5 $$ \overline{Q}_{i} = \frac{{F_{i} }}{{F_{i} + F_{j} }}\overline{Q}_{p} \;\;\;\;{\text{where}}\;\;\;F_{i} = \left( {\frac{{\pi r_{0}^{4} }}{8\mu L}} \right)_{i} $$

Here $$i$$ and $$j$$ are the two daughter branches, and $$\overline{Q}_{{\text{p}}}$$ denotes the mean flow in the parent vessel (Qureshi et al. 2019), and for terminal branches, we assume that $$R_{P} $$ and $$R_{D}$$ are initially equivalent, i.e., $$R_{{\text{P}}} = R_{{\text{D}}} = R_{{\text{T}}} /2$$. The determination of $$C$$ requires fitting an exponential decay function to the change in $$P_{{{\text{MPA}}}}$$ from systole to diastole (Qureshi et al. 2019). Total proximal and distal resistance in the network is calculated from the sum of the resistances in parallel, while the total compliance in the network is calculated as using circuit theory of capacitators in parallel (Qureshi et al. 2019).

In the experimental data set used here, Mulchrone et al. (2019) previously observed an asymmetric distribution of obstruction with the majority of blockage in the left lung. Because less contrast then flowed into the left lung, imaging resolution was poorer on this side. To account for signal loss, the flow into the left lung in CTEPH models was recalculated as $\tilde{Q}_{{{\text{LPA}}}} \left( t \right) = Q_{{{\text{MPA}}}} \left( t \right) - Q_{{{\text{RPA}}}} \left( t \right)$ and then used to calculate the left lung Windkessel resistance and compliance distributions.

### Numerical simulation and model calibration

The system Eqs. (1)–(4) are solved numerically using Richtmyer’s two-step Lax–Wendroff. To achieve convergence to a steady state, simulations are conducted until the least square error between consecutive pressure cycles is less than 1e-8. The spatial and temporal step sizes are 0.125 (cm) and $\approx$ 1e-4 (s), respectively.

**Fig. 3:**
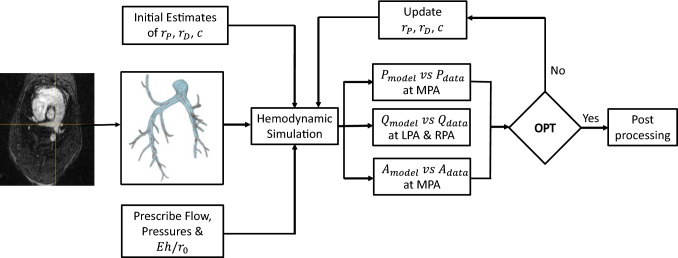
Schematic of model calibration process for Windkessel parameters with experimental pressure, area, and flow data

Windkessel parameters were inferred based on the set of calibration data $\left\{ {P_{{{\text{sys}}}} , P_{{{\text{dia}}}} , A_{{{\text{sys}}}} , A_{{{\text{dia}}}} , Q_{{{\text{LPA}}}} \left( t \right), Q_{{{\text{RPA}}}} \left( t \right)} \right\}$ at baseline and $\left\{ {P_{{{\text{sys}}}} , P_{{{\text{dia}}}} , A_{{{\text{sys}}}} , A_{{{\text{dia}}}} , \tilde{Q}_{{{\text{LPA}}}} \left( t \right), Q_{{{\text{RPA}}}} \left( t \right)} \right\}$ at CTEPH. Given the limited data and numerous Windkessel elements (i.e., $R_{P} , R_{D}$, and $C$ for each terminal branch), the Windkessel parameters were scaled by the same set of scaling factors ${\varvec{\theta}}_{{{\text{wk}}}} = \left\{ {r_{{\text{P}}} ,r_{{\text{D}}} , c} \right\}$ using weighted, nonlinear least squares (Qureshi et al. 2019). We defined a cost function in which the model predictions are matched to the calibration data (and denoted by a superscript $c$) by minimizing:

6 $$ S\left( {\varvec{\theta}} \right) = \mathop \sum \limits_{j} \left( {P_{j}^{c} - P_{j} \left( {{\varvec{\theta}}_{{{\text{wk}}}} } \right)} \right)^{2} + \mathop \sum \limits_{j} \left( {(A_{j}^{c} - A_{j} \left( {{\varvec{\theta}}_{{{\text{wk}}}} } \right)} \right)^{2} + \frac{1}{N}\mathop \sum \limits_{k} \mathop \sum \limits_{i = 1}^{N} \left( {Q_{k}^{c} \left( {t_{i} } \right) - Q_{k} \left( {t_{i} ;{\varvec{\theta}}_{{{\text{wk}}}} } \right)} \right)^{2} $$

where $$P_{j}^{c} $$ is the calibration pressure for $$j = {\text{sys}},\;{\text{dia}}$$, $$P_{j} ({\varvec{\theta}}_{{{\text{wk}}}} {)}$$ is computed pressure, $$A_{j}^{c}$$ is the calibration area, and $$A_{j} ({\varvec{\theta}}_{{{\text{wk}}}} {)}$$ is the computed area. For the time-series data terms,* N* is the length of the time vector spanning one cardiac cycle, $$Q_{k}^{c} \left( {t_{i} } \right) $$ denotes calibration flow data for $$k = {\text{LPA}},\;{\text{ RPA}}$$, and $$Q_{k} (t_{i} ;\theta_{{{\text{wk}}}} {)}$$ is the computed time-series flow at time $$t_{i}$$. We used the function *lsqnonlin* in MATLAB to identify the global minimum for our objective function. This was accomplished through ten randomized initial values. All ten initial guesses successfully converged to the same value, and the optimization process halted when the parameters ceased to change by more than 1e-6, as specified. The calibration process is shown in Fig. 3.

### Wall shear stress

The TAWSS and OSI were computed as follows:

7 $$ {\text{TAWSS}} = \frac{1}{T}\mathop \smallint \limits_{0}^{T} \left| {\tau \left( {x,t} \right)} \right|{\text{d}}t\;\;\;\;{\text{where}}\;\;\;\;\tau \left( {x,t} \right) = \mu \left( {\frac{{\partial u\left( {x,t} \right)}}{{\partial r\left( {x,t} \right)}}} \right)_{r = R} $$

and

8 $$ {\text{OSI}} = 0.5 \left( {1 - \frac{{\frac{1}{T} \left| {\mathop \smallint \nolimits_{0}^{T} \tau \left( {x,t} \right){\text{d}}t} \right|}}{{\frac{1}{T}\mathop \smallint \nolimits_{0}^{T} \left| {\tau \left( {x,t} \right)} \right|{\text{d}}t}}} \right) $$

Throughout the arterial network. Here, $\tau \left( {x,t} \right)$ (dyne/cm^2^) is the spatially- and temporally-dependent arterial WSS, and $T$ (s) is the cardiac cycle length. Note that whereas TAWSS and OSI in the large, extralobar arteries are straightforward to compare from baseline to CTEPH, the number and size of intrapulmonary arteries detectable in the imaging, and thus represented in the 1D model, vary from baseline and CTEPH. To enable comparison from baseline to CTEPH, we report TAWSS and OSI averaged over all intralobar arteries for each subject.

### Statistical test and analysis

We hypothesized a priori that the hemodynamic parameters and output quantities will either increase or decrease with CTEPH based on clinical understanding of the disease. We opted to invoke this assumption in our statistical test and therefore used a paired one-sided t-test (in Excel) to assess significance with *p* < 0.05. Pearson correlation was used based on relative changes between the model outputs from baseline to CTEPH to better understand disease progression among the different subjects.

## Results

### Model calibration

**Fig. 4:**
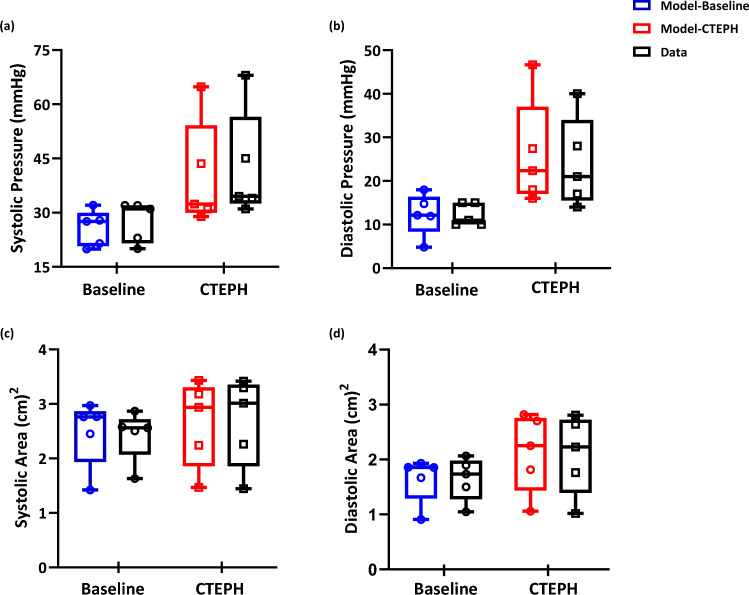
Comparison between measured data and model prediction. **a** Systolic pressure, **b** diastolic pressure, **c** systolic area, and **d** diastolic area

**Fig. 5:**
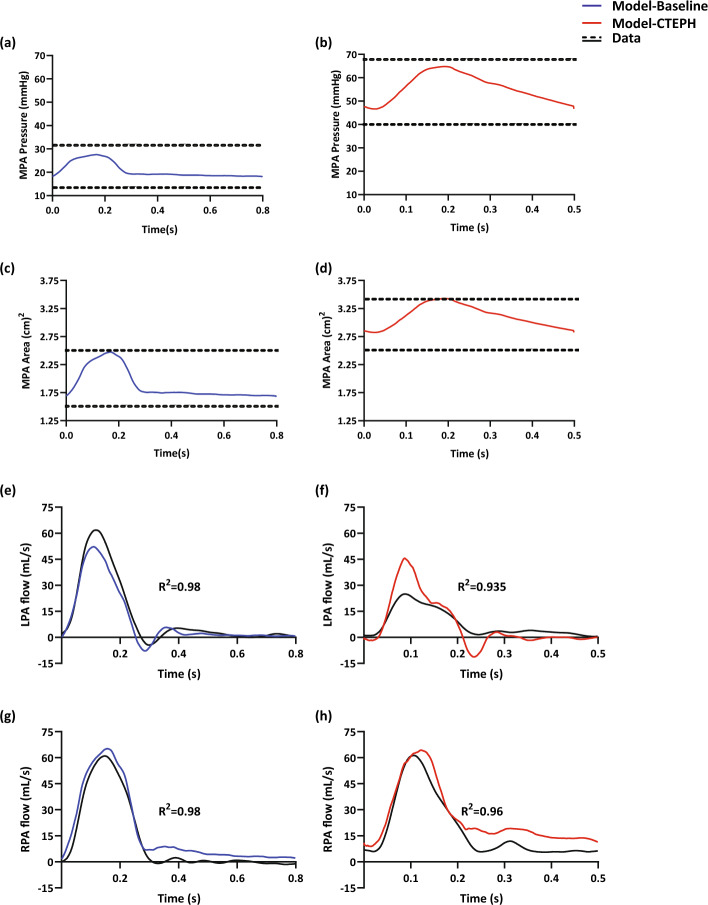
Measured hemodynamics and model predictions for a representative subject. **a**, **b** Model pressure predictions (solid lines) compared to measured systolic and diastolic pressure (dashed lines). The model predicts blood pressure values with $\pm$ 3 mmHg of the data, given the inferred values of stiffness and Windkessel parameters in both baseline and CTEPH. **c**, **d** The predicted areas values from the model (solid lines) and the measured systolic and diastolic pressures at MPA (dashed lines). Panels **e**–**h** show LPA flow and RPA flow, respectively, from measurements (black) and model predictions (blue, left at baseline and red, right with CTEPH). In this animal, a notable decrease in mean flow at LPA was observed after CTEPH induction reduction of approximately 25%. Conversely, an increase in mean flow of approximately 45% was observed in the right lung

Model fits to scalar calibration data $\left\{ {P_{{{\text{sys}}}} , P_{{{\text{dia}}}} , A_{{{\text{sys}}}} , A_{{{\text{dia}}}} } \right\}$ at baseline and CTEPH are shown in Fig. 4. Following the development of CTEPH, all subjects had increased systolic and diastolic pressures as expected (Fig. 4a and b). Four out of five subjects had increased systolic and diastolic areas. We attribute the one exception to low image resolution at baseline, which led to a non-significant group increase with CTEPH (Fig. 4c and d). Agreement between model simulations and the time-series experimental measurements $\left\{ {Q_{{{\text{LPA}}}} \left( t \right), Q_{{{\text{RPA}}}} \left( t \right)} \right\}$ was assessed by their *R*^2^ value. Table 1 summarizes the average and standard deviation of relative error between measured data and model estimations for systolic and diastolic pressures and areas at baseline and CTEPH, as well as *R*^2^ values for LPA and RPA. As previously observed for this experimental data set (Mulchrone et al. 2019), all subjects had a reduced cardiac output and significant asymmetries in flow distribution after CTEPH. Representative model results and comparison to experimental data are shown for a single subject at baseline and after CTEPH (Fig. 5).

**Table 1:** Relative error for systolic and diastolic pressures and areas and *R*^2^ values for LPA and RPA flows between measured data and model estimations for all subjects in baseline and CTEPH conditions (average (AVG) and standard deviation (SD) shown)

	Relative error in pressure (%)				Relative error in area (%)				$R^{2}$ for dynamic flow			
	*Diastolic*		*Systolic*		*Diastolic*		Systolic		LPA		RPA	
	Base	CTEPH	Base	CTEPH	Base	CTEPH	Base	CTEPH	Base	CTEPH	Base	CTEPH
AVG	− 14	7.0	− 6.1	− 6.0	− 0.7	2.2	0.7	− 1.0	0.95	0.92	0.98	0.96
SD	47	5.7	5.3	1.9	9.0	3.5	7.6	1.9	0.05	0.03	0.01	0.01

### Pulmonary arterial network properties

**Fig. 6:**
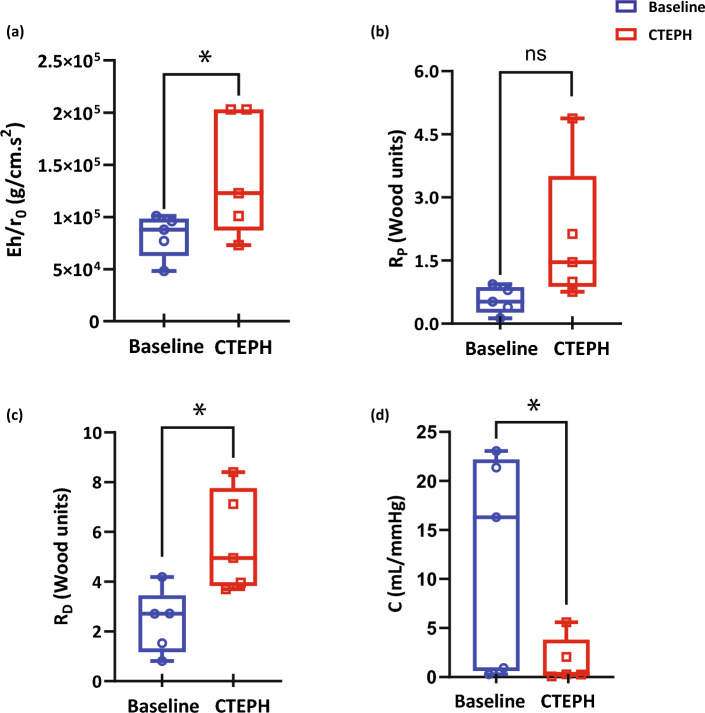
Changes in the material stiffness terms (**a**), proximal resistance (**b**), distal resistance (**c**), and compliance (**d**) from baseline (blue) to CTEPH (red). *p < 0.05

Pulmonary arterial stiffness was analytically computed for each subject using $\left\{ {P_{{{\text{sys}}}} , P_{{{\text{dia}}}} , A_{{{\text{sys}}}} , A_{{{\text{dia}}}} } \right\}$ in Eq. (6). As shown in Fig. 6a, CTEPH significantly increased $\frac{Eh}{{r_{0} }}$ (*p* = 0.02), which can be attributed to the combination of structural and load-dependent stiffening. CTEPH increased Windkessel total proximal resistance $R_{{\text{P}}}$ (*p* = 0.07) and significantly increased total distal resistance $R_{{\text{D}}}$ (*p* = 0.04) (Fig. 6b and c) and significantly decreased Windkessel total vascular compliance $C$ (*p* = 0.032) (Fig. 6d).

### Wall shear stress

**Fig. 7:**
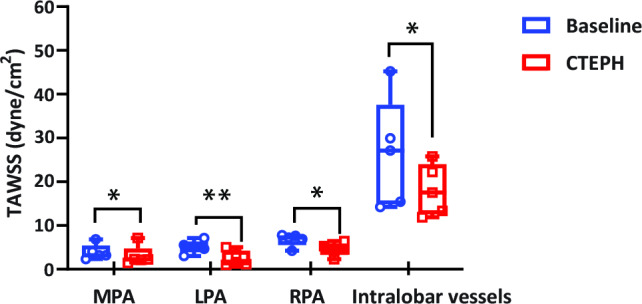
MPA, LPA, RPA, and intralobar arterial TAWSS under baseline (blue) and CTEPH (red) conditions; *n* = 5. *p < 0.05, **p < 0.01

**Fig. 8:**
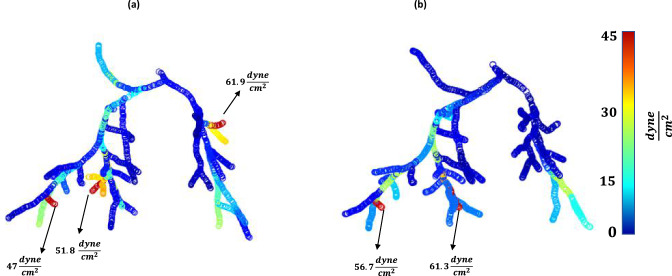
Three-dimensional map of TAWSS distribution across the arterial network for a representative animal at baseline (**a**) and CTEPH (**b**). This map demonstrates the changes in TAWSS that occurred in response to CTEPH induction throughout different segments of the network

With the model predictions of $\left\{ {P\left( {x,t} \right),A\left( {x,t} \right), Q\left( {x,t} \right)} \right\}$, changes in TAWSS with CTEPH were computed (Fig. 7). In four out of five subjects, TAWSS decreased at the MPA and RPA; in all subjects, TAWSS decreased in the LPA and most intralobar arteries. One subject exhibited an increase in MPA TAWSS (by $\approx$ 0.2(dyne/cm^2^)), and one subject exhibited an increase in RPA TAWSS (by$\approx$ 1 (dyne/cm^2^)) following CTEPH. On average, there was a significant decrease in TAWSS in the MPA, LPA, RPA, and intralobar arteries (*p* = 0.027, *p* = 0.005, *p* = 0.048, and *p* = 0.022, respectively). A three-dimensional map displaying the distribution of TAWSS across the arterial network for a representative subject at baseline and CTEPH is shown in Fig. 8. At baseline, TAWSS values ranged from 1.12 to 61.9 (dyne/cm^2^) with a mean value of 15.02 (dyne/cm^2^), whereas after CTEPH, TAWSS decreased, ranging from 0.34 to 61.4 (dyne/cm^2^) with a mean value of 12.8 (dyne/cm^2^).

**Fig. 9:**
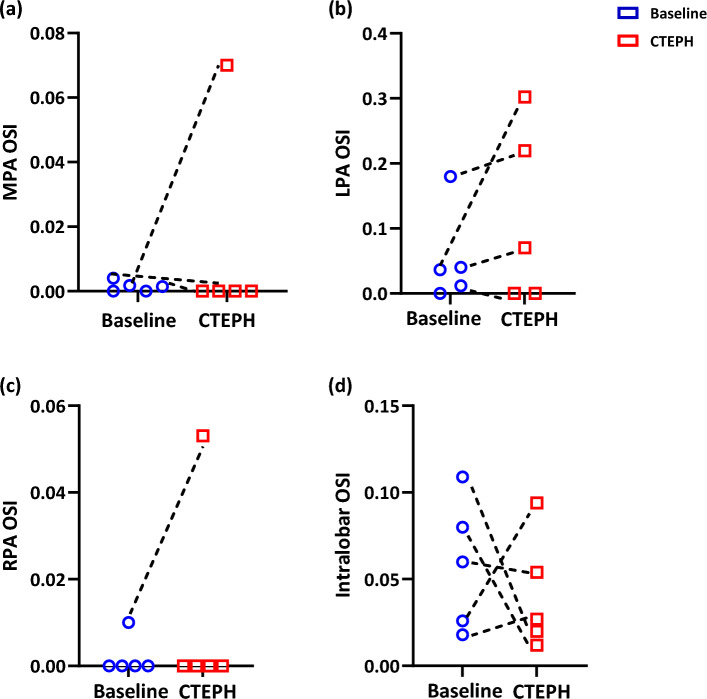
MPA (**a**), LPA (**b**), RPA (**c**), and intralobar arteries (**d**) OSI under baseline (blue) and CTEPH (red) conditions; *n* = 5

Model flow simulations also enabled computation of OSI distribution. With CTEPH, OSI increased and decreased in the extralobar (MPA, LPA, and RPA) and intralobar arteries without a consistent pattern (Fig. 9). For several subjects, MPA and RPA OSI values were essentially zero.

To investigate the impact of the velocity profile on TAWSS and OSI, we used $\gamma = 2$ (Poiseuille flow), $\gamma = 5,$ $\gamma = 7,$ and $\gamma = 9$. For a representative subject at baseline, TAWSS was on the order of 9 dyne/cm^2^ for $\gamma = 2$ and steadily increased to approximately 15, 20, and 24 dyne/cm^2^ for $\gamma = 5,7,$ and 9, respectively. In CTEPH, TAWSS was approximately 7 dyne/cm^2^ for $\gamma = 2$ and increased in a similar manner to baseline, with $\gamma = 5, 7,$ and 9 providing TAWSS values of approximately 13, 16, and 20 dyne/cm^2^, respectively. Values of OSI varied slightly at baseline and CTEPH for these values of $\gamma$.

**Fig. 10:**
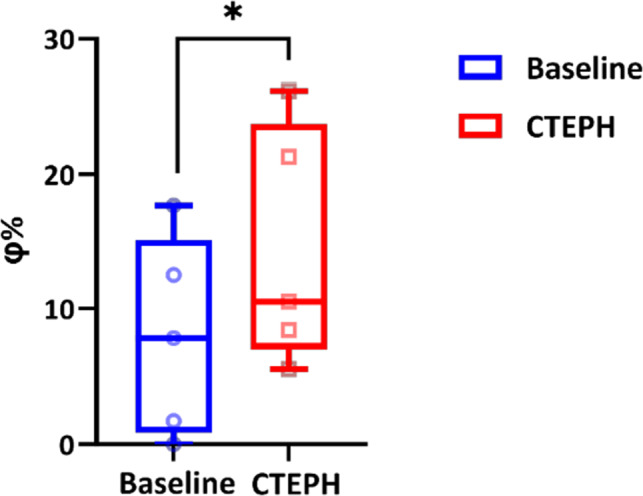
Percentage of vessels that have TAWSS < 5 (dyne/cm^2^) and OSI > 0.05 at baseline (blue) and CTEPH (red) conditions; *n* = 5. *p < 0.05

To better understand the hemodynamic impact of CTEPH, we investigated changes in a combined metric using the spatial distribution of TAWSS and OSI. We identified regions of the network with both low TAWSS (< 5 dyne/cm^2^) and high OSI (> 0.05); the TAWSS cutoff was based on the previous literature (Li et al. 2009), and the OSI cutoff was based on the average OSI for all vessels across all subjects. As shown in Fig. 10, the percentage of regions that met this criterion, $\varphi$, significantly increased with CTEPH (*p* = 0.023).

### Correlation analysis

**Fig. 11:**
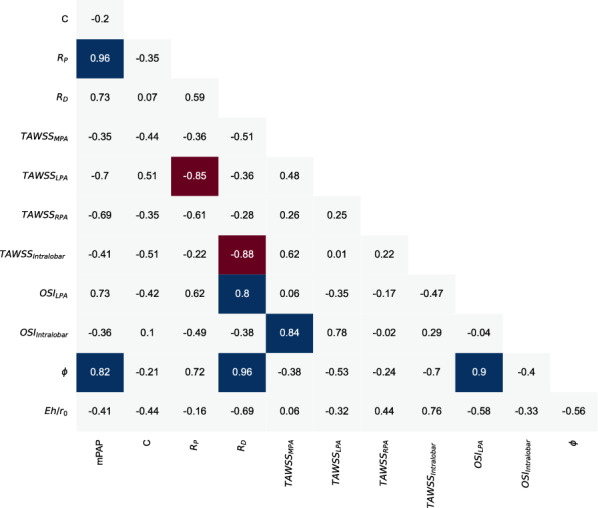
Correlation analysis for model outputs based on the relative changes from baseline to CTEPH for each subject. All Pearson correlation coefficients shown with values > 0.80 and < − 0.80 highlighted

To analyze the subject-specific parameter value changes from baseline to CTEPH conditions, we performed Pearson correlation analysis. We excluded MPA and RPA OSI from the analysis since most of the values were essentially zero. Correlations values are shown in Fig. 11, with those above 0.80 and below − 0.80 highlighted as strong correlations. We found strong positive correlations between mPAP and proximal resistance, MPA TAWSS and intralobar OSI, $\varphi$ and mPAP, $\varphi$ and distal resistance, and $\varphi$ and LPA OSI. Conversely, we observed strong negative correlations between proximal resistance and LPA TAWSS, and between distal resistance and intralobar TAWSS.

## Discussion

In this study, we used an image-based nonlinear one-dimensional CFD model to estimate pulmonary arterial hemodynamic features in the extralobar and intralobar regions. Measuring hemodynamics in the intralobar vessels would be difficult, if not impossible, to measure experimentally and may be clinically relevant to disease progression, which motivates the current work.

Based on an existing set of imaging data under baseline and CTEPH conditions, we developed subject-specific 1D arterial network models. We used an efficient multi-target optimization scheme for calibration that incorporated experimentally measured MPA systolic and diastolic pressures and areas, as well as dynamic flow data at the LPA and RPA. Our approach yielded simulation results with excellent agreement with measured data and estimated pulmonary arterial stiffness, TAWSS, and OSI distribution in both baseline and CTEPH. Pulmonary arterial stiffness can drive upstream (i.e., right ventricular (Liu et al. 2022)) and downstream (i.e., pulmonary capillary (Wang and Chesler 2011)) remodeling to accelerate disease progression. Moreover, changes in WSS are relevant to the growth or dissolution of thrombi (Sakariassen et al. 2015). Thus, these model results allow for a more comprehensive understanding of the effects of CTEPH, enhancing our ability to study this condition and potentially improve patient care and treatment strategies.

### Modeling and optimization

Hemodynamics modeling is a powerful tool for investigating hypothesized mechanisms of pulmonary vascular disease development and progression. There has been limited application of these tools to CTEPH in comparison with other forms of pulmonary hypertension, such as pulmonary arterial hypertension (PAH) (Clipp and Steele 2009; Zambrano et al. 2018; Dong et al. 2021). The use of subject-specific modeling has the potential to provide clinicians with a better understanding of patient-specific disease states, optimizing clinical decision-making, and ultimately enabling personalized medicine. Several computational studies calibrated their models using existing experimental data. Zambrano et al. (2018) developed subject-specific computational models of pulmonary vascular hemodynamics for both a PAH patient and a healthy individual. The parameters of the model, including Windkessel parameters ($R_{P} ,R_{D},$ and $C$) for each terminal vessel in the outflow branches, were adjusted using in vivo measurements of pressure from RHC. Additionally, the linearized stiffness E of the large arteries was adjusted based on the relationship between pressure and diameter from MRI. Clipp and Steele (2009) generated 1D CFD models of the pulmonary arterial networks of lambs, incorporating considerations such as geometry, compliance, structured tree boundary conditions, and respiration. Similar to Zambrano et al. (2018), parameters describing the boundary conditions in the Clipp et al. study were inferred by comparing the computed inlet pressure waveform to experimental data. In this study, we not only used pressure calibration data (systolic and diastolic) to infer boundary conditions but also used area (systolic and diastolic) and flow (time-series) calibration data, enhancing model accuracy and reliability. In this study, we used one set of scaling parameters, ${\varvec{\theta}}_{wk}$, and applied it to all terminal branches. Despite the relative simplicity of this approach, model calibration was reasonably accurate, as illustrated in Fig. 5 and Table 1. Certain subjects in both the baseline and CTEPH groups exhibited nonzero MPA diastolic flow, including the representative animal showcased in Fig. 5. Our model enforces mass conservation, and considering the non-simultaneous acquisition of measured flow data at the extralobar vessels, there may be a discrepancy between our model’s diastolic flow predictions and the actual measured data, as depicted in Fig. 5g and h.

There are few studies that use computational modeling to estimate hemodynamics in CTEPH. Spazzapan et al. (2018) created segmentations of pulmonary arteries from postoperative chest CT scans of three CTEPH patients, including a focal stenosis in the pre-surgical geometry, and then performed three-dimensional flow simulations to analyze hemodynamics. They observed an asymmetrical flow split, which also occurred in our animal model of CTEPH. Colebank et al. (2021) developed 1D CFD models of pulmonary arterial networks based on computed tomography imaging in controls with no PH. They simulated four different CTEPH disease cases to test physiological hypotheses related to remodeling. Tsubata et al. (2023) performed 3D CFD simulations to model subject-specific data from healthy subjects, CTEPH patients, and CTEPH patients treated with balloon pulmonary angioplasty. However, neither paper had an abundance of baseline and CTEPH data from individual subjects. A novel aspect of our study is that we used paired data sets of baseline and CTEPH conditions, which enables a subject-specific interpretation of CTEPH progression using correlation methods.

### Pulmonary arterial parameters

Pulmonary hypertension is known to increase pulmonary arterial stiffness, which is measured in clinical and preclinical studies using a wide range of direct and indirect methods. Su et al. (2017) used wave intensity analysis to indirectly measure pulmonary arterial stiffness in 10 healthy subjects and 21 subjects with pulmonary hypertension (11 with PAH and 10 with CTEPH). They reported that the wave reflection index in these subjects was significantly higher than in healthy controls. We analytically determined the mechanical properties of the PAs using systolic and diastolic pressure and a linear wall mechanics model. In contrast, the previous studies (Qureshi et al. 2019) required parameter estimation to determine pulmonary arterial stiffness. Colebank et al. (2021) included a larger arterial tree (226 branches) as well as a model of the microcirculation through the structured tree, which also had a radius-dependent stiffness. In contrast, our study uses only a simplified representation of arteries from imaging data, reducing stiffness variability in proximal vasculature. Colebank et al. employed an exponential stiffness model, with values similar to those reported in Fig. 5a. Ebrahimi et al. (2021) simulated PAH and CTEPH conditions using a transmission-line model of the whole pulmonary circulation. These authors showed that a combination of vascular narrowing and changes in stiffness could replicate flow heterogeneity seen in clinical CTEPH. Similar to our findings, Ebrahimi et al. concluded that stiffness alone cannot account for the pressure–flow dynamics seen in clinical CTEPH cases, and a combination of distal vascular remodeling, clot presence, and stiffness is likely contributors to disease progression. As shown in Fig. 6a and consistent with the literature, we observed vessel stiffening after CTEPH induction, which likely contributes to disease progression (Sun and Chan 2018).

Pulmonary arterial stiffening in CTEPH is a combination of load-dependent stiffening and structural stiffening (Wang and Chesler 2011; Pewowaruk et al. 2021). That is, the increase in pulmonary vascular resistance due to CTEPH increases pressure and thus increases the operating point of arteries on the nonlinear pressure–diameter curve (Wang and Chesler 2011), called load-dependent stiffening. In addition, proliferative and fibrotic changes in the pulmonary arterial wall cause structural remodeling that increases vessel stiffness (Kim et al., 2000). The combination of increased resistance and stiffness elevates right ventricular afterload and contributes to the development of the right heart failure (Wang and Chesler 2011). Qureshi et al. (2019) used a model similar to ours to study hemodynamic changes in normoxic and hypoxic mice. Their Windkessel resistance increased in hypoxia while Windkessel compliance decreased. Figure 6b-c illustrates an increase in Windkessel proximal and distal resistance with CTEPH, primarily attributable to the obstruction and narrowing of pulmonary arteries and arterioles, respectively. Additionally, as depicted in Fig. 6d, a decrease in Windkessel compliance occurs with CTEPH, likely due to a combination of load-dependent and structural stiffening in the distal arteries and arterioles.

### Flow and shear stress

Changes in the flow of blood within the lungs are believed to play a crucial role in the onset and worsening of pulmonary vascular diseases (Clark and Tawhai 2018). Here, changes in flow magnitude and distribution were observed in both lungs for all subjects from baseline to CTEPH. The change in distribution with CTEPH, in particular the left–right asymmetry, is due to uneven microspheres distribution that can be attributed to the catheter tip being oriented predominantly toward the LPA (Mulchrone et al. 2019), which may not reflect the clinical disease state. Due to these flow changes as well as artery dilation, our 1D model estimates that WSS decreases throughout the arterial network with CTEPH. Note, whereas the magnitude of TAWSS depends on the shape of the velocity profile (i.e., the $\gamma$ value), the trend in TAWSS changes from baseline to CTEPH does not. The previous 1D fluid dynamic studies (Alastruey et al. 2008; Clipp and Steele 2009; Spilker and Taylor 2010) used power-law velocity profiles with $\gamma$ = 9. Based on our analysis of 2D phase-contrast images at the MPA, $\gamma$ = 5 provides a better fit.

While different types of PH have different mechanisms of disease, they share some similarities. PAH and CTEPH both cause intimal thickening and inflammation. There exist potential shared pathways behind the two diseases, including endothelial cell dysfunction and distal pulmonary artery remodeling (Humbert 2010). It has been suggested that *in situ* thrombosis in the lungs, resulting from primary arteriopathy and endothelial dysfunction akin to that observed in PAH, might be a contributing factor to the development of CTEPH (Egermayer and Peacock, 2000; Peacock et al. 2006). Due to these similarities, we compare our results with some previous studies in PAH.

Clinical studies that use image-based methods to compute WSS in individuals have shown that those with PH exhibit lower WSS than normotensive subjects (Barker et al. 2015; Terada et al. 2016). Modeling studies have found the same result. Zambrano et al. (2021) performed 3D image-based, computational fluid dynamics simulations, in which interactions between blood flow and wall deformation were included and predicted that subjects with PAH have lower TAWSS than controls. Tang et al. (2012) used the same approach as Zambrano et al. (2021) and similarly predicted lower mean WSS in the proximal arteries in the PAH group. From patient-specific 3D computational fluid dynamics simulations in CTEPH, Tsubata et al. (2023) observed lower systolic WSS in CTEPH patients in comparison with controls as well as to patients after balloon pulmonary angioplasty treatment. Our results are consistent with the results of these image-based and computational studies, as shown in Fig. 7.

At the MPA, we found that TAWSS significantly decreased following CTEPH in four of the five subjects; we attribute the one exception to poor image quality at baseline that likely increased the estimated arterial network area. A significant reduction in LPA TAWSS was observed for all subjects in our study, which can be attributed to a dramatic drop in flow to the left lung due to the severity of the obstruction. This finding is consistent with the suggestion that WSS reduction in the proximal PAs is an indicator of PAH disease severity (Yang et al. 2019). At the RPA, TAWSS decreased in all subjects except one (not the same subject for which MPA TAWSS increased). Wall shear stress is directly proportional to the flow rate and its magnitude. Therefore, due to the significant reduction in flow in the left lung compared to the right lung in CTEPH, the decrease in TAWSS RPA was less dramatic than in the LPA (Fig. 7).

In the intralobar arteries, TAWSS is higher compared to the extralobar arteries in both baseline and CTEPH conditions. This observation is consistent with Pillalamarri et al. (2021) who found that TAWSS increased with increasing distance from the heart based on a computational investigation of patient-specific pulmonary hemodynamics across 32 subjects in distinct WHO groups of PH. The Pillalamarri et al. (2021)study included one subject with CTEPH, in whom TAWSS decreased in the distal part of the network, consistent with our results.

The biological consequences of decreased WSS in the pulmonary circulation include altered endothelial cell signaling that drives vasoconstriction and the production of pro-inflammatory factors (Allen et al. 2023). Decreased TAWSS may also contribute to the synthesis and accumulation of collagen in the arterial wall and proliferation of smooth muscle cells (Ryu et al. 2022). This pulmonary arterial remodeling can result in a stiffer and less compliant pulmonary artery network (Friesen et al. 2019), which further exacerbates the severity of PH and can lead to the right heart failure.

### Oscillatory shear index

Like TAWSS, OSI can drive changes in endothelial cell structure and function that affect disease progression. In endothelial cells, high OSI is associated with increased reactive oxygen species, which can drive vasoconstriction and inflammation (Peiffer et al. 2013). In the large extrapulmonary arteries, especially the MPA and RPA, OSI magnitude was quite small at baseline and only became somewhat larger with CTEPH. MRI-based measurements of MPA flow, which were used as input boundary conditions, showed no evidence of reverse flow in two animals at baseline and four at CTEPH. As a consequence, MPA OSI was essentially zero in these animals, and downstream OSI values were also near zero. In contrast, Tsubata et al. (2023) found a significant increase in proximal artery OSI in CTEPH subjects compared to controls. Their OSI magnitudes were on average much larger than ours (0.05 verses 0.35). Bartolo et al. (2022) used a 1D framework similar to ours to compute OSI in a human pulmonary arteriovenous network and found OSI magnitudes similar to ours, which suggests that the 1D framework and/or the assumed velocity profile affects OSI calculations. One limitation of the OSI prediction in our 1D model is that secondary flow phenomena are not included. Another limitation is the reverse flow near the wall in the MPA may not be captured by the MRI-based measurement due to limitations in spatial resolution. Nevertheless, our approach does offer insight into how OSI changes during disease development.

In the intralobar arteries, there was no clear pattern regarding the impact of CTEPH on OSI (Fig. 9d). To further investigate temporal abnormalities in intralobar flow dynamics, we considered the combination of TAWSS and OSI (Fig. 10) represented by the dimensionless parameter $\varphi$. Tsubata et al. (2023) suggested that the combination of decreased WSS and increased OSI can contribute to thrombogenicity. Our results indicate that the percentage of vessels that had low TAWSS (< 5dyne/cm^2^) and high OSI (> 0.05) increased from baseline to CTEPH in all subjects. When the vascular endothelium experiences a decrease in wall shear stress and an increase in OSI, it inhibits the production and release of nitric oxide, a potent vasodilator (Terada et al. 2016). As a result, the abnormal flow dynamics as seen in our study may be linked to impaired vasodilation and vasoconstriction in the pulmonary arterioles.

### Correlation analysis

The importance of the novel parameter $\varphi$ is supported by the strong correlations with mPAP, distal resistance, and LPA OSI in individual subjects from baseline to CTEPH conditions. The correlation between mPAP and proximal resistance is intuitive and reflects increased afterload; in contrast, the correlation between MPA TAWSS and intralobar OSI is surprising since a higher mean flow proximally should increase mean flow (and thus attenuate negative flow) distally. We interpret the negative correlation between proximal resistance and LPA TAWSS to indicate more severe left-sided obstruction. Analogously, we interpret the negative correlation between distal resistance and intralobar TAWSS to indicate more severe CTEPH disease. Applied to larger data sets, this correlative analysis could yield novel indicators of disease severity and progression.

### Limitations

Several limitations exist in our study, notably the small sample size, which contributed to heterogeneity. Especially in correlation analysis, having a larger sample size and working in higher dimensions would enable stronger conclusions. Limited image resolution in some subjects, especially one subject at baseline and a different subject after CTEPH, resulted in challenges extracting accurate subject-specific arterial networks and changes in those arterial networks with CTEPH. In the subject with image quality issues at baseline, the result was a relatively simplified arterial tree. However, as highlighted by Colebank et al. (2019), the omission of a few branches in a large network model does not significantly impact the resulting fluid dynamics. In the subject with poor image quality with CTEPH, we had difficulty distinguishing whether a region of the left lobe with low contrast was the result of occlusion or limited image resolution. Based on the CTEPH flow data for this subject, which was significantly reduced in the left lung, we interpreted it as the former. On that basis, we did not segment those sections with poor resolution and instead replaced the downstream vasculature with a Windkessel model parameterized to reflect the measured flow.

Since time-series pressure data were not recorded for either baseline or CTEPH conditions, we calibrated our model based on the scalar values of systolic and diastolic pressures (as well as scalar values of systolic and diastolic MPA area and time-series LPA and RPA flow). The inclusion of time-series MPA pressure data would increase model fidelity. Even though our model captures measured data with an acceptable accuracy, our inability to exactly match the measured data can be attributed to (1) noisy data, (2) an imperfect model framework, or (3) an insufficient number of free parameters for inference.

We determined MPA arterial stiffness using a simple, linear pressure–area relationship. Uniaxial mechanical testing was conducted on some pulmonary artery tissues from some subjects after terminal studies but could not compare these *ex vivo* experimental results to the calibrated values of MPA arterial stiffness. We attribute this to (1) a lack of comprehensive data including reference areas and (2) insufficient data to match *in vivo* pressure–area to *ex vivo* stress–strain, which is important given pulmonary arterial nonlinear elasticity.

The 1D model assumes axisymmetric flow in the axial direction only and does not account for secondary flow phenomena such as separation and recirculation. It is unclear how these secondary flows would contribute to OSI magnitude and distribution; a 3D fluid structure interaction (FSI) model could be used to assess the impact of our 1D approach on OSI results. We assumed Womersley-like flow with a power-law radial distribution for all arteries and held the power fixed at *γ* = 5. However, the range of Womersley number in the models was 3–28, and for these lower Womersley values, the *γ* value should be lower. As a consequence, in the small arteries, which will have lower Womersley numbers, TAWSS is overestimated. The impact on OSI is difficult to predict a priori; comparison to Tsubata et al. (2023) suggests that OSI is underestimated in our model. Adapting *γ* to local diameter in the 1D CFD model would increase model complexity and solution time but increase accuracy.

## Conclusion

The impact of CTEPH on hemodynamic and vascular mechanical parameters that cannot be measured directly was examined using a subject-specific 1D CFD modeling framework. We calibrated the model to RHC- and MRI-derived measurements of pressure, area, and flow from a previously published large animal model of CTEPH. Our analysis revealed that CTEPH increased arterial stiffness and decreased TAWSS, and that the combination of decreased TAWSS and increased OSI in intralobar arteries correlates with disease severity. Our experimental–computational framework shows potential as a patient-specific simulator of both extralobar and intralobar pulmonary hemodynamics within the context of pulmonary hypertension. By leveraging advanced modeling techniques with model calibration, we can gain a more wholistic understanding of CTEPH, which could ultimately lead to more targeted interventions that address the specific needs of individuals with CTEPH.
